# A Case of Severe Acute Pancreatitis Secondary to COVID-19 Infection in a 30-Year-Old Male Patient

**DOI:** 10.7759/cureus.11718

**Published:** 2020-11-26

**Authors:** Haidar Alwaeli, Mahvesh Shabbir, Mali Khamissi Sobi, Karar Alwaeli

**Affiliations:** 1 Medical Education and Simulation, Richmond University Medical Center, New York, USA; 2 Family Medicine, A+ Urgent Care Center, Atlanta, USA; 3 Pharmacology and Therapeutics, Roseman University of Health Sciences, Henderson, USA

**Keywords:** covid-19, coronavirus, pancreatitis, coronavirus disease 2019, covid-19-associated coagulopathy, stroke and covid-19, covid-19 respiratory failure, prophylactic and therapeutic anticoagulation, covid-19 pneumonia

## Abstract

A 30-year-old male with no significant medical history presented to the emergency department with complaints of fever, two days of intermittent abdominal pain, dry cough, nausea, vomiting, four days of diarrhea, and worsening dyspnea. Initial evaluation revealed a fever of (102.5 F) and tachycardia (114/min) with hypoxia (SaO2: 84% on room air) and bilateral wheezing on lung auscultation. X-ray of the chest revealed bilateral and peripheral ground-glass and consolidative pulmonary opacities. CT scan of the abdomen was notable for interstitial edema, mild inflammatory changes, and homogenous enhancement of the pancreatic parenchyma. His COVID-19 test came positive, and he was admitted to the intensive-care unit. He was managed symptomatically, and improvement in his clinical condition was observed after three days of admission. This case highlights a possible association between Severe Acute Respiratory Syndrome Coronavirus 2 (SARS-CoV-2), abdominal pain secondary to acute pancreatitis, and the need for meticulous clinical evaluation in patients presenting with gastrointestinal complaints.

## Introduction

Pancreatitis is an inflammatory process involving the pancreas, which can be subdivided into two main categories: acute and chronic [1]. Acute pancreatitis is a common disease with a wide range of clinical presentation, from its low-risk symptoms to a severe and acute process of multiple organ failure and increased risk of morbidity and mortality [2]. Gallstones remain the most common cause of acute pancreatitis. However, other causes of both acute and chronic pancreatitis include heavy alcohol use, viral or parasitic infections, cancer, and trauma [3]. With the recent COVID-19 pandemic, we have seen a surge in hospital visits due to gastrointestinal symptoms. Gastrointestinal involvement, such as abdominal pain, diarrhea, nausea, and vomiting, have all been reported recently in the literature [4]. We report a rare case of COVID-19 infection with acute respiratory distress and pancreatitis.

## Case presentation

A 30-year-old Middle Eastern male with a body mass index of 21.4 presented with fever, dry cough, nausea, abdominal pain, vomiting, four days of diarrhea, and worsening dyspnea. The patient reports that for the past week he has had watery diarrhea and has had a decrease in appetite. Two days prior, he began having intermittent abdominal pain that initially felt like excessive bloating pains but now progressed to be constant. The pain was located in the epigastric area and radiates to the back. Additionally, he reports the use of melatonin (2 mg PO QHS) for the past two days, for symptoms of insomnia. He denies a history of alcohol use, smoking, jaundice, pruritis, hemoptysis, melena, or hematochezia. Physical examination of the patient showed fever (102.5 F) and tachycardia (114/min) with hypoxia (SaO2: 84% on room air) and bilateral wheezing on lung auscultation. Abdominal examination revealed moderate distention with diffuse tenderness to superficial palpation, with no peritonitis signs. Admission laboratory workup is summarized in Table 1. Laboratory findings were pertinent for elevated lipase and amylase (lipase/amylase elevation > three times the upper limit of normal) with a slight elevation in serum aspartate aminotransferase (AST) and alanine aminotransferase (ALT). A posteroanterior chest X-ray revealed bilateral and peripheral ground-glass and consolidative pulmonary opacities. Hepatitis viral serology for A, B, and C, and human immunodeficiency virus (HIV) were all negative. Moreover, the patient tested positive for COVID-19 by a real-time reverse transcriptase-polymerase chain reaction (RT-PCR) assay.

**Table 1:** Clinical laboratory findings

Laboratory evaluation at the time of admission (reference ranges of laboratory data are included in parentheses)	
Hematocrit (36.7-46.4%)	37.8%
Hemoglobin (12.5-15.7 g/dL)	14.5 g/dL
Total leukocyte count (3,800-11,000)	5,480/ uL
Band neutrophils	2%
Total neutrophils	51%
Lymphocytes	38%
Monocytes	9%
Eosinophils	2%
Platelets (140,000-400,000/μL)	352,000/μL
Total bilirubin (≤ 1.2 mg/dL)	0.9mg/dL
Direct bilirubin (≤ 0.25 mg/dL)	0.12 mg/dl
Serum aspartate aminotransferase (AST) (13-35 IU/dL)	67
Alanine aminotransferase (ALT) (7-35 IU/dL)	88 IU/L
Alkaline phosphatase (64-300 IU/dL)	198 U/dL
Albumin (3.5-5.0 g/dL)	3.6 g/dL
Triglyceride (≤ 150 mg/dL)	133 mg/dL
Lipase (≤ 200 IU/L)	1,022 IU/L
Amylase (20-110UI/L	151 IU/L
Serum calcium (9.0-10.8 mg/dL)	8.4 mg/ dL

**Figure 1:**
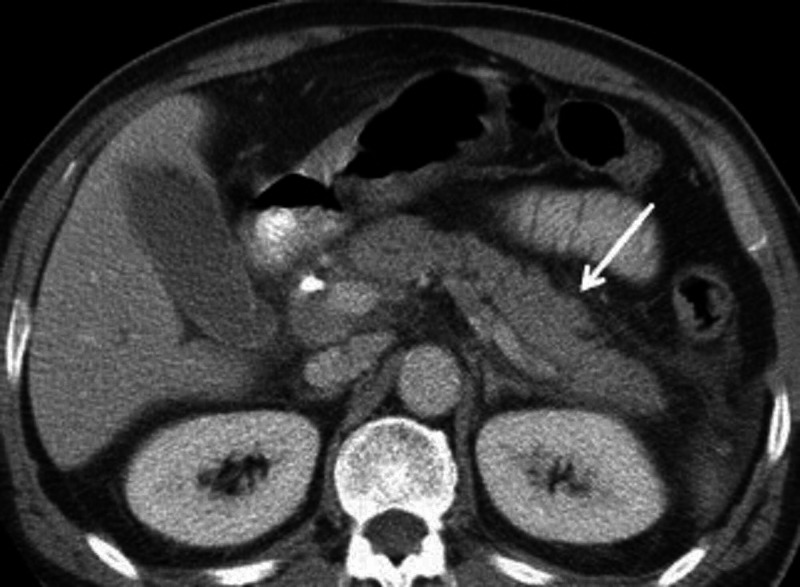
Abdominal CT scan showing interstitial edema of the pancreas with the homogenous enhancement of the pancreatic parenchyma and mild inﬂammatory changes (fat stranding)

The patient was diagnosed with acute respiratory distress syndrome (ARDS) with severe acute pancreatitis secondary to COVID-19 infection and as a result, was admitted to the intensive-care unit. The patient was managed and treated with intravenous fluids, fasting, and total parenteral nutrition, antibiotics, antiemetics, and analgesics. Axial CT image of the abdomen was notable for interstitial edema of the pancreas with the homogenous enhancement of the pancreatic parenchyma and mild inflammatory changes (fat stranding) surrounding the pancreas (Figure 1). Supportive therapy continued, and improved clinical condition was observed after three days of treatment. The patient’s oxygen saturation improved drastically (97% on room air) and no longer required oxygen therapy. The patient was discharged from the hospital after clinical improvement of symptoms.

## Discussion

In late December 2019, the WHO China County office reported a number of pneumonia cases of unknown cause observed in Wuhan City, China. This, of course, resulted in major investigations and the confirmation of the novel coronavirus as the primary cause of an outbreak. The World Health Organization announced an official name for the disease on February 11, 2020. This important milestone marked the beginning of a global emergency and pandemic. To date, the SARS-CoV-2 has continued to have a catastrophic impact on international relations, global health, global network in production, social networking, economy, and approximately 50 million people worldwide [5, 6]. Typically, patients infected with COVID-19 present with classical presentations of respiratory symptoms such as a cough, dyspnea, fever, and pharyngitis. However, we continue to discover new extrapulmonary manifestations. The severity of COVID-19 is typically combined with a set of comorbid conditions such as diabetes, hypertension, cardiovascular disease, advanced age, and obesity. Furthermore, infected patients can present with atypical symptoms related to the gastrointestinal system. One study, in particular, indicated that COVID-19 patients experienced gastrointestinal symptoms such as diarrhea (24.2%), anorexia (17.9%), and nausea (17.9%) [7].

About 10% of pancreatitis cases are thought to be directly caused by infectious microorganisms such as parasites, bacteria, and viruses [8]. Viral pancreatitis has been widely reported in medical literature to be most commonly caused by mumps, viral hepatitis, coxsackieviruses, echoviruses, and mycoplasma [9]. The mechanism of the relation with the development of pancreatitis associated with COVID-19 infection is unknown and multifactorial. For example, several factors may contribute to the development of pancreatitis, including premature activation and autodigestion of pancreatic enzymes and microcirculatory disturbance of the pancreas. Theoretical explanations for the association between COVID-19 and pancreatitis include cellular overexpression of the angiotensin-converting enzyme 2 (ACE2), a molecular target for SARS-CoV-2 [10]. Additionally, extrapulmonary manifestations of fulminant myopericarditis secondary to COVID-19 infection have been reported [11]. The reported case demonstrated the possible correlation between COVID-19 and nonspecific gastrointestinal symptoms, life-threatening ARDS, and myopericarditis. Based on our case above, the established diagnosis of pancreatitis in this patient appears to be idiopathic due to the patient's lack of comorbid conditions, history of alcohol abuse, or trauma. It is worth noting that there is a possible correlation between COVID-19 infection and acute pancreatitis. However, there is insufficient evidence to link COVID-19 directly to acute pancreatitis as a causative agent, and further studies need to be conducted to evaluate the possible correlation.

## Conclusions

This case of a 30-year-old male patient demonstrates that COVID-19 infection can cause nonspecific gastrointestinal symptoms and acute pancreatitis. The diagnosis of COVID-19 was established by utilizing PT-PCR. Additionally, abnormal lipase, amylase enzyme levels were obtained, and CT scan findings were all indicative of acute pancreatitis. We encourage medical practitioners to carefully and meticulously evaluate gastrointestinal symptoms in patients with active COVID-19 patients in order to prevent delays in diagnosis and treatment.
